# Effects of Reforestation on the Structure and Diversity of Bacterial Communities in Subtropical Low Mountain Forest Soils

**DOI:** 10.3389/fmicb.2018.01968

**Published:** 2018-08-21

**Authors:** Yu-Te Lin, William B. Whitman, David C. Coleman, Chih-Yu Chiu

**Affiliations:** ^1^Biodiversity Research Center, Academia Sinica, Taipei, Taiwan; ^2^Department of Microbiology, University of Georgia, Athens, GA, United States; ^3^Odum School of Ecology, University of Georgia, Athens, GA, United States

**Keywords:** 16S rRNA genes, soil bacterial community, long-term reforestation, coniferous, broadleaved

## Abstract

Reforestation with different tree species could alter soil properties and in turn affect the bacterial community. However, the effects of long-term reforestation on bacterial community structure and diversity of subtropical forest soils are poorly understood. In the current study, we applied error-corrected barcoded pyrosequencing to characterize the differences in the soil bacterial community in a low mountain, subtropical forest subjected to reforestation. The communities were sampled in the summer and winter from a native broadleaved forest (BROAD-Nat) and two adjacent coniferous plantations, a *Calocedrus formosana* forest of 80 years (CONIF-80) and a *Cunninghamia konishii* forest of 40 years (CONIF-40). The soil bacterial communities among three forest types were dominated by *Acidobacteria* and *Alphaproteobacteria*. The distribution of abundant genera among communities was different. Based on the Shannon diversity index, the bacterial alpha diversity of CONIF-40 community was significantly higher than that in the CONIF-80 and BROAD-Nat soils. In both of the coniferous plantations, the soil bacterial diversity in summer was also higher than that in winter. Distribution of some abundant phylogenetic groups, *K*-shuff and redundancy analysis of beta diversity among communities showed that the bacterial structure of three soil communities differed between two seasons. These results suggest that seasonal differences influence the diversity and structure of bacterial soil communities and that the communities remain different even after a long period of reforestation.

## Introduction

Soil bacteria play an important role in soil processes, including mineralization of organic matter and biogeochemical cycling of carbon and nitrogen (Bardgett et al., 2008; Chatterjee et al., 2008; Burton et al., 2010). The soil bacterial community can be influenced by soil properties (Stres et al., 2008; Ushio et al., 2008; Lauber et al., 2009). Different plant species can also result in distinct soil bacterial communities (Oh et al., 2012). Similarly, other environmental factors can play important roles, such as litter quality and root exudates (Quideau et al., 2001; Grayston and Prescott, 2005; Xu et al., 2008). Seasonal differences in temperature and precipitation also alter soil bacterial communities. For example, soil microbial phospholipid fatty acid (PLFA) composition associated with organic matter decomposition was significantly affected by temperature differences (Zhou et al., 2016). Multiple factors, including temperature, resulted in the complex responses of bamboo soil bacterial composition and diversity across altitudinal gradients (Lin et al., 2015). Previous studies also indicated that precipitation could affect bacterial communities indirectly by changing the soil moisture, which may have resulted in composition differences between perhumid forest sites with similar elevations but different precipitation (Lin et al., 2014).

The disturbances caused by forest management and other land uses are another important factor affecting soil microbes. They can alter soil characteristics and then influence the diversity and structure of the microbial community (Hartmann et al., 2014). For instance, the abundances of *Proteobacteria* and *Firmicutes* were significantly correlated with the disturbances of soil compaction associated with logging (Hartmann et al., 2014). Rainforest conversion to rubber plantation significantly increased the abundance of *Chloroflexi* in the bacterial community, while the most abundant phylum, *Acidobacteria*, was largely unchanged (Kerfahi et al., 2016). The proportion of some abundant bacterial groups differed between communities of native and regenerated perhumid montane forest soils (Lin et al., 2011b).

In the current study, we analyzed the differences of soil bacterial communities at a subtropical low mountain forest subjected to reforestation. This forest ecosystem is mainly covered by natural broadleaved forest. In the same region, the natural forest was replaced by a *Calocedrus* plantation 80 years ago and a *Cunninghamia* plantation 40 years ago. In a previous study using 16S ribosomal ribonucleic acid (rRNA) gene clone libraries and Sanger sequencing, we found that the reforestation with conifers altered the structure of the bacterial community and increased bacterial diversity (Lin et al., 2011a). In addition, another study indicated that the microbial biomass, PLFA content and soil enzymatic activities were higher in the summer than in the winter (Chang et al., 2016). However, the PLFA method cannot provide information about microbial populations at the genus or family level (Papadopoulou et al., 2011), and the low number of sequences from clone library only provided data on the abundant members of the community (Caporaso et al., 2011), leaving a gap in our understanding of the effects of season and disturbance on the soil bacterial community at this ecosystem. Hence, we applied the barcoded pyrosequencing method to obtain a more comprehensive survey of the soil bacterial communities. Compared to the clone library, the pyrosequencing method is less labor intensive and can provide between 4,500 and 52,000 unique sequences or operational taxonomic units (OTUs) in soils (Acosta-Martínez et al., 2010). Thus, it quickly provides much more comprehensive information about the microbial community structure. We hypothesized that both the change in management and seasonal differences would increase the soil bacterial diversity and alter the abundance of some bacterial taxa. The first objective of this study was to determine the soil bacterial diversity and structure in natural broadleaved forest compared to the coniferous plantation soils. Our second objective was to compare the effects of season on these soil bacterial communities.

## Materials and Methods

### Site Description and Soil Sampling

This study was conducted at Lienhuachi Experimental Forest, a subtropical low mountain area (about 700 m.a.s.l.) in central Taiwan (23°54′N, 120°54′E). It is a long-term ecological study site of low altitude forest ecosystem and encompasses 461 ha, with 261 ha covered by evergreen natural hardwood forest. Approximately 50% of the region has been replanted by coniferous plantations, including *Calocedrus formosana* and *Cunninghamia konishii*. The mean annual precipitation is about 2,200 mm. Nearly 90% of which occurs between March and September. The mean annual temperature is about 21°C (30°C in July and 10°C in January) (Lee et al., 2008). Soil samples were collected in July, 2010 (summer) and February, 2012 (winter). A native broadleaf forest (BROAD-Nat), and secondary coniferous *Calocedrus* plantation of 80 years (CONIF-80) and *Cunninghamia* plantation of 40 years (CONIF-40) were included for sampling. Each vegetation type was sampled at four (winter) or five (summer) 50 m × 50 m plots established 50 m apart along transect lines. The plots were marked to facilitate subsequent sample collections at the same location. After removing the surface litter, three subsamples at each plot were collected with a soil auger, 8 cm in diameter and 10 cm deep and pooled. The soil samples were passed through a 2 mm sieve, and a portion was stored at 4°C for soil biochemical assays. The remainder of the samples was freeze-dried and stored in -20°C for DNA extraction. The soils in sampling sites were moderately well-drained and classified as Typic Dystrudept (U.S. Soil Taxonomy) (Jien et al., 2009). The soils were strongly acidic, and the pH values of the surface soils ranged from 3.6 to 3.8. Other characteristics of each soil site are described in Chang et al. (2016).

### DNA Extraction and Amplification of 16S rRNA Genes and Pyrosequencing

The soil communities DNA from 0.25 g of soil from each plot were extracted using the PowerSoil^TM^ Soil DNA Isolation kit (MoBio Industries, Carlsbad, CA, United States) in accordance with manufacturer’s instructions. The V1 to V2 regions of the bacterial 16S rRNA gene were amplified using 27F and 338R primers (Lane, 1991). Polymerase chain reactions (PCRs) were performed as described previously (Lin et al., 2015). Secondary PCR (3 cycles rather than 20) was carried out to add the different barcodes for each sample (Lin et al., 2015). The unique and error-correcting barcodes facilitated sorting of sequences from a single pyrosequencing run (Hamady et al., 2008). Products were then purified using the PCR clean up system (Viogene BioTek Corp., New Taipei City, Taiwan). The qualities and concentrations of the purified barcoded PCR products were determined using a NanoDrop Spectrophotometer (Thermo Fisher Scientific). Amplicon pyrosequencing was performed by Mission Biotech (Taipei, Taiwan) using a 454/Roche GS-FLX Titanium Instrument (Roche, Branchburg, NJ, United States).

### Pyrosequence Analyses

All the sequences were processed through the Ribosomal Data Project (RDP) pyrosequencing pipeline^1^ (RDP release 11.5; release date: 2016.09.30). Sequences were assigned to each sample by recognition of the barcode and trimmed to remove the barcodes, primers, and linker. The pyrosequences were then filtered, and those that did not have Ns, were more than 200 bp in length, were free of chimeras using Uchime (Edgar et al., 2011), and had quality scores >25 were used for further analyzes. The trimmed and filtered sequences used for the subsequent analyses were submitted to the Sequence Read Archives with the accession number of SRP136858. Taxonomic information was analyzed using the Naïve Bayesian rRNA classifier of the RDP with a confidence cutoff of 80% (Wang et al., 2007). OTUs were formed with an evolutionary distance of 0.03. Singletons that appeared only once in the data set were removed. To avoid measurement differences due to differences in sample size for estimation of the alpha and beta diversity (Roesch et al., 2007; Will et al., 2010), the sample size of each replicate was normalized to 1800 sequences by random selection from larger samples. Although this procedure reduced the number of rare OTUs analyzed, it was justified because the goals of these studies were to compare the effects of season and vegetation on the bacterial communities and not to capture all OTUs. To estimate bacterial alpha diversity, Shannon diversity indexes (Shannon and Weaver, 1963) were calculated based on the Complete Linkage Clustering data for OTUs with an evolutionary distance of 0.03. The “Venn” command of Mothur program (Schloss et al., 2009) was applied to visualize the shared OTUs among bacterial communities. The abundant genera and genus-level groups of three forest soil bacterial communities were identified with the Naïve Bayesian rRNA classifier of the RDP. The differences in distribution between the seasons were analyzed with one way ANOVA. The heat map was plotted with Heatmapper (Babicki et al., 2016). Community comparisons were further performed using *K*-shuff analysis (Jangid et al., 2016). Correlations (Spearman rank correlation ρ) between resemblance matrices of bacterial composition based on the distribution of OTUs, diversity, and soil properties were assessed using RELATE as implemented in the PRIMER V6 software (Clarke and Gorley, 2006). A permutation test (999 permutations) was applied to evaluate significance. The resemblance matrix was derived from normalized data. Under the null hypothesis that there is no relation between the two similarity matrices, ρ will be approximately zero. Redundancy analysis was carried out by using the vegan package in R v.3.2.1 to determine the relationship between the distribution of OTUs of bacterial community and soil properties.

## Results

### Bacterial Community Composition

**FIGURE 1:**
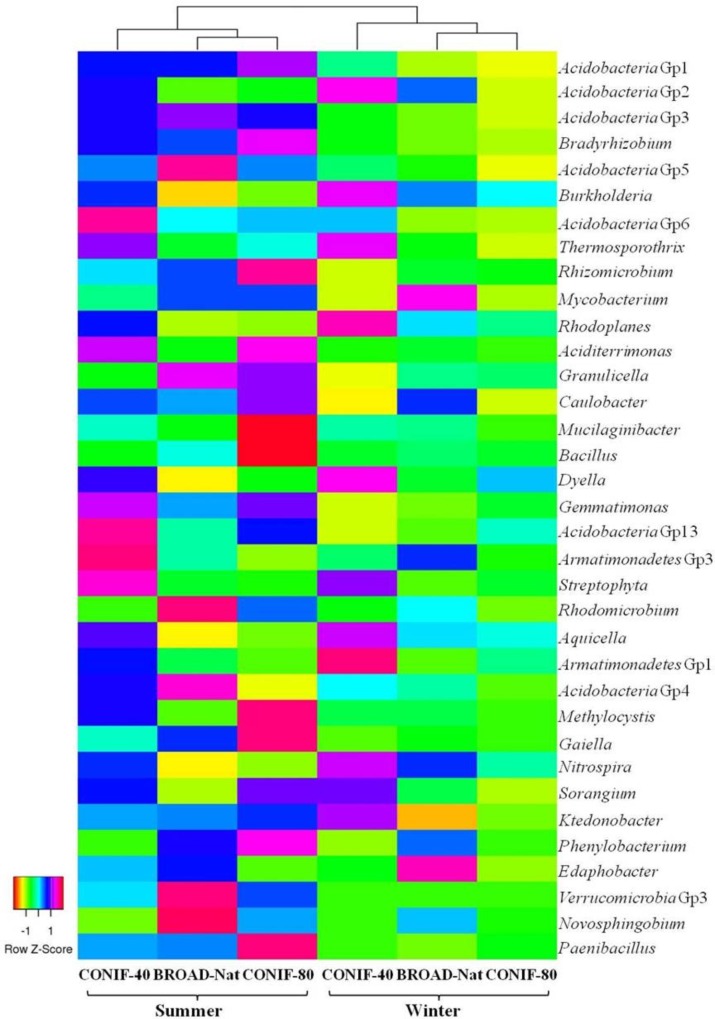
Heat map of the 35 most abundant genera or genus-level groups of three forest soil communities. Dendrogram of the Euclidean distances between the bacterial communities is shown at the top.

Using barcoded pyrosequencing, the 16S rRNA gene sequences were retrieved from three forest soils in summer and winter to investigate the bacterial composition. About 12,300 to 24,000 pyrosequence reads were obtained from each soil communities (**Supplementary Table S1**). These sequences were dominated with *Acidobacteria* (42–48%) and *Proteobacteria* (30–33%) (**Supplementary Figure S1**). *Actinobacteria* was the third most abundant phyla, but accounted for less than 6% of the sequences. The proportion of other phyla, such as *Bacteroidetes*, *Chloroflexi*, and *Firmicutes*, were all less than 4% (**Supplementary Figure S1**). The Gp1, 2, and 3 were the most abundant groups of *Acidobacteria*, and the relative abundance of Gp2 was higher in winter (**Table 1** and **Figure 1**). In contrast, *Acidobacteria* Gp3 was more abundant in summer. Within the *Proteobacteria*, *Alphaproteobacteria* was the most abundant group (17–22%), and *Bradyrhizobium* of the *Rhizobiales* was the most abundant genus, especially in the summer. The *Beta*-, *Gamma*- and *Delta-proteobacteria* were all only about 2–5% of sequences among the three communities in both seasons. In summary, although the same bacterial genera and genus-level groups were abundant under all the conditions examined, the relative abundances of some phylogenetic groups varied significantly with season.

**Table 1:** The relative abundance (%) of some abundant genera and genus-level groups of three forest soil bacterial communities^a^.

Affiliation	Summer			Winter			% of all reads
	BROAD-Nat	CONIF-80	CONIF-40	BROAD-Nat	CONIF-80	CONIF-40	
*Acidobacteria* Gp1	24.1	22.9	20.2	20.1	22.4	20.5	21.8
*Acidobacteria* Gp2^∗^	7.5	6.9	9.7	14.7	11.1	14.3	10.3
*Acidobacteria* Gp3	6.8	5.3	5.3	4.6	4.7	4.3	5.2
*Bradyrhizobium*^∗^	2.2	2.4	2.0	1.7	1.8	1.6	2.0
*Acidobacteria* Gp5	1.9	1.1	1.1	1.1	0.9	1.1	1.2
*Burkholderia*^∗^	0.7	1.2	2.9	4.3	4.3	4.9	1.1
*Acidobacteria* Gp6	0.9	0.8	1.2	0.9	1.0	1.1	1.0
*Thermosporothrix*^∗^	0.6	0.6	0.7	0.8	0.8	1.0	0.8
*Rhizomicrobium*	0.7	0.8	0.5	0.7	0.8	0.4	0.8
*Mycobacterium*	0.6	0.5	0.3	1.2	0.3	0.2	0.6
*Rhodoplanes*^∗^	0.5	0.5	1.4	1.7	1.7	2.4	0.5
*Aciditerrimonas*	0.3	0.5	0.5	0.5	0.5	0.3	0.5
*Granulicella*	0.7	0.5	0.2	0.4	0.5	0.1	0.4
*Caulobacter*	0.4	0.4	0.3	0.6	0.4	0.2	0.4
*Mucilaginibacter*	0.2	0.5	0.2	0.3	0.3	0.3	0.4
*Bacillus*	0.3	0.5	0.1	0.3	0.3	0.2	0.3
*Dyella*	0.2	0.4	0.8	0.7	1.3	1.3	0.3
*Gemmatimonas*^∗^	0.2	0.3	0.3	0.1	0.2	0.0	0.2
*Acidobacteria* Gp13	0.2	0.2	0.3	0.1	0.3	0.1	0.2
*Armatimonadetes* Gp3	0.1	0.1	0.2	0.2	0.2	0.1	0.2
*Streptophyta*	0.1	0.1	0.2	0.1	0.1	0.2	0.1
*Rhodomicrobium*	0.3	0.1	0.0	0.2	0.0	0.1	0.1
*Aquicella*^∗^	0.1	0.2	0.4	0.5	0.5	0.6	0.1
*Armatimonadetes* Gp1	0.1	0.1	0.1	0.1	0.2	0.2	0.1
*Acidobacteria* Gp4	0.2	0.0	0.1	0.1	0.1	0.1	0.1
*Methylocystis*	0.0	0.2	0.1	0.1	0.1	0.1	0.1
*Gaiella*	0.1	0.2	0.1	0.1	0.1	0.0	0.1
*Nitrospira*^∗^	0.1	0.1	0.2	0.4	0.4	0.4	0.1
*Sorangium*	0.0	0.1	0.1	0.1	0.1	0.1	0.1
*Ktedonobacter*	0.1	0.1	0.1	0.0	0.1	0.1	0.1
*Phenylobacterium*	0.1	0.1	0.0	0.1	0.0	0.0	0.1
*Edaphobacter*	0.1	0.0	0.1	0.2	0.0	0.0	0.1
*Verrucomicrobia* Gp3	0.2	0.1	0.1	0.0	0.0	0.0	0.1
*Novosphingobium*	0.2	0.1	0.0	0.1	0.0	0.0	0.1
*Paenibacillus*	0.1	0.1	0.0	0.0	0.0	0.0	0.1


*Affiliations with an asterisk indicate a significantly different (*P* < 0.05) in distribution between seasons analyzed with one way ANOVA. ^a^The data was based on the results from the Naïve Bayesian rRNA classifier of the RDP. The total reads included in table were about 50% of normalized data.*

Similar conclusions were obtained by *K*-shuff analysis of the soil bacterial communities (**Table 2**). Within summer or winter, the community structures were significantly different between the three forest soils except for the comparison of CONIF-80 and CONIF-40 in summer. Moreover, the soil bacterial community of each forest type also differed significantly between two seasons. However, in all cases, the differences were relatively small, with *K*_cf_ values of less than 0.005.

**Table 2:** Statistical significance (*P*-value) of differences among forest soil communities using *K*-shuff analysis^a^.

Season	Forest	Summer			Winter		
		CONIF-80	CONIF-40	BROAD-Nat	CONIF-80	CONIF-40	BROAD-Nat
Summer	CONIF-80	–	0.06	**0.01**	**0.01**	0.21	**0.02**
	CONIF-40	0.000092	–	**0.01**	**0.01**	**0.01**	0.11
	BROAD-Nat	0.001825	0.001833	–	**0.02**	**0.01**	**0.01**
Winter	CONIF-80	0.001372	0.001280	0.004077	–	**0.01**	**0.03**
	CONIF-40	0.001320	0.001228	0.004026	0.000052	–	**0.01**
	BROAD-Nat	0.000350	0.000441	0.002356	0.001701	0.001670	–


*The lower parts of the matrix were the K_*CF*_ values between forest soil communities. ^*a*^Values in bold are P ≤ 0.05.*

### Bacterial Community Diversity

**FIGURE 2:**
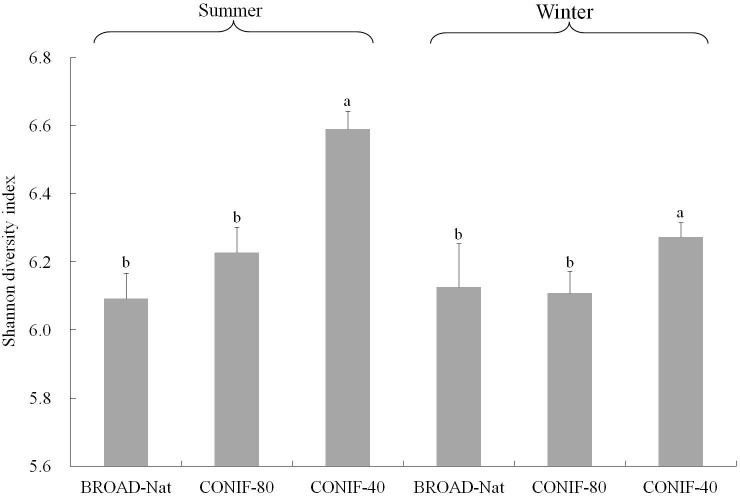
Shannon diversity index of three forest soil bacterial communities. OTUs were calculated at the 3% evolutionary distance. Bars of the same season with different letters indicate significant differences at *P* ≤ 0.05 analyzed with one way ANOVA. The bacterial diversity of the same forest types between two seasons was not significantly different.

Although the major taxa present in all three forest soils were similar, the bacterial alpha diversity among three communities was different. Based on the Shannon diversity indexes calculated from the OTUs formed at an evolutionary distance <0.03 (about 97% sequence similarity), the CONIF-40 forest soil community was more diverse than both the CONIF-80 and BROAD-Nat communities in both the summer and winter (**Figure 2**). While the diversity of both the CONIF-40 and CONIF-80 forest communities appeared to be higher in the summer than the winter, this difference was not significant (**Figure 2**). Rarefaction curves supported these conclusions, and the slopes of the CONIF-40 curves were higher than the other communities in each season (**Supplementary Figure S3**). In addition, the rarefaction curves for the BROAD-Nat communities were nearly identical in both seasons.

### Bacterial Community Comparison and Relationship With Soil Properties

While the same genera and genus-level groups were abundant in all the bacterial communities, there were significant variations in the relative abundance of some specific groups (**Table 1**). To examine the differences in more detail, the distributions of the OTUs with more than 10 reads in the normalized data set were visualized in Venn diagrams (**Supplementary Figure S2**). These OTUs represented 53–65% of the total number of reads. The minimum number of reads of 10 was chosen because the rarefaction curves indicated that the communities had not been completely sampled (**Supplementary Figure S3**), and OTUs with fewer reads might be found in only one community by chance even if it was evenly distributed throughout the samples. In all comparisons, either by management or season, nearly 70% of the OTUs were only found in one condition. Moreover, statistical analyses of the *K*-shuff results indicated that the communities were significantly different in both seasons (**Supplementary Table S2**). These results strongly supported the conclusion that the communities varied greatly with both season and management.

**FIGURE 3:**
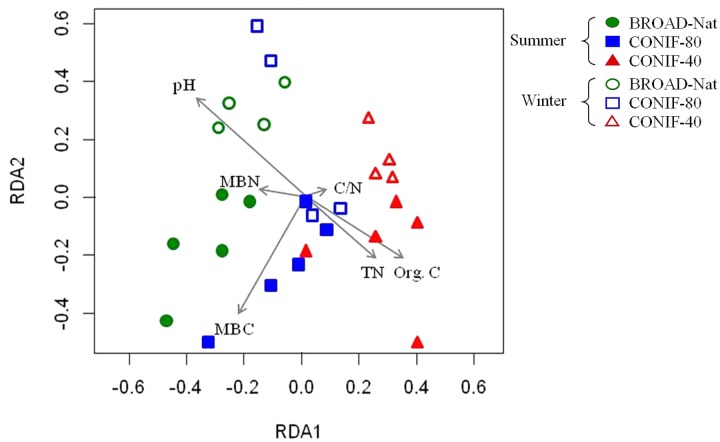
Redundancy analysis of bacterial community based on the distribution of OTUs among three forest soils in different seasons. Org. C, organic carbon; TN, total nitrogen; C/N, organic C/TN ratio; MBC, microbial biomass carbon; MBN, microbial biomass nitrogen.

Based on the RELATE analysis, soil properties, including soil pH and organic carbon, were significantly correlated with the alpha diversity of bacterial communities (**Table 3**). Redundancy analysis was further performed based on the distribution of shared OTUs and the soil properties. The soil communities between two seasons, especially those in BROAD-Nat forests, formed separate clusters (**Figure 3**). This result supported the analyses of abundant genus-level groups and OTUs (**Table 1**), which reached similar conclusions. In addition, the results of **Supplementary Table S2** also supported the conclusion that replicate samples mostly clustered together, as in **Figure 3**. The microbial biomass carbon was also positively associated with the BROAD-Nat and CONIF-80 forest communities in summer. The Org. C and TN showed positive associations with the summer CONIF-40 community, and the pH was positively associated with BROAD-Nat and CONIF-80 forest communities in winter (**Figure 3**).

**Table 3:** Correlations of bacterial community composition based on the distribution of OTUs and Shannon diversity index with soil properties using the RELATE in the PRIMER V6 software^a^.

Soil Properties	Correlation	
	Composition	Diversity
pH	0.19	**0.30**
Organic C	0.17	**0.18**
Total N	0.01	0.06
C/N ratio	0.01	-0.14
Microbial biomass C	0.09	0.06
Microbial biomass N	0.05	-0.08


*^*a*^Values were Spearman’s correlation coefficient (ρ)between bacterial composition, diversity and soil properties. Values in bold are P ≤ 0.05*.

## Discussion

In the present study, the three bacterial communities were dominated by two phyla, *Acidobacteria* and *Proteobacteria*. With the other three less abundant groups, *Actinobacteria*, *Bacteroidetes*, and *Firmicutes*, they comprised more than 90% of the sequences in each of the communities. Using pyrosequencing, similar results have also been observed in other bacterial communities of forests (Lauber et al., 2009; Nemergut et al., 2010), grasslands (Will et al., 2010), and agricultural systems (Acosta-Martínez et al., 2008). These results indicated that a variety of soils were mainly composed of similar dominant bacterial groups, although the relative abundances of the taxa varied between the study sites.

The bacterial structure in natural broadleaved forest soils differed from those in secondary coniferous plantations. Several studies have shown that land use is one of the important factors to alter the soil bacterial structure (Deng et al., 2016; Wang et al., 2017). Our previous study at higher elevations also showed the bacterial structure changes between a natural broadleaved forest and reforested cedar plantation (Lin et al., 2017). Forest management in tree species changes could result in differences in litter chemistry and influence soil properties and microbial communities (Ushio et al., 2010). Soil nutrient availability is correlated with the bacterial community (Hofmann et al., 2016).

Our results revealed that the community diversity of coniferous CONIF-40 and perhaps the CONIF-80 plantation soils was higher than that in the original BROAD-Nat forest soils. Previous studies in different forest ecosystem also revealed that the disturbance of forest soils increased the diversity of microbial communities (Lin et al., 2011a, 2017). The disturbance of reforestation could disturb soil water content and nutrient conditions, which in turn could affect bacterial diversity (Jangid et al., 2011). Although the apparent diversity in the coniferous plantation soils was higher in the summer than the winter, this increase was not significant. Temperature and soil moisture are important factors that affect the soil bacterial community. A landscape-scale study in Scotland revealed that the soil bacterial composition was related to variation in precipitation (Nielsen et al., 2010). Differences in soil water content due to the temporal variability affected the soil bacterial alpha diversity (Rasche et al., 2011; Lauber et al., 2013). In this study site, precipitation is mainly between March and September. The temperature in summer could be up to 30°C and only 10°C in winter. Lower temperature and precipitation in winter could alter microbial activity (Sowerby et al., 2005) and shape the communities (Romanowicz et al., 2016), resulting in lower bacterial diversity in winter.

Using clone library method, a previous study at the same site also revealed the community differences between hardwood and coniferous plantations. However, the *Proteobacteria* comprised more than half of sequences, and the *Gammaproteobacteria* dominated within the *Proteobacteria* (Lin et al., 2011a). The results were quite different from the present study. In the present study, the soil samples were recollected and were different from those in the previous one. Besides sampling time, sequencing method could result in this difference. About 150 to 170 sequences per sample were used in the previous study or far less than the present one. The low number of sequences may have missed some parts of community, and 2,000 reads have been suggested as necessary to characterize soil microbial communities (Caporaso et al., 2011). In addition, an abundant *Stenotrophomonas*-related OTU was present in the previous study (Lin et al., 2011a). Less than five pyrosequences of this genus was retrieved in the present study. Thus, the specific cloning method may also have contributed to the differences.

In this study, *Acidobacteria* predominated in the communities of the three forests. The abundance is in accordance with findings of other studies of the composition of soil bacterial communities conducted in various environments, such as forest (Lauber et al., 2009), grassland (Will et al., 2010), and agricultural soils (Janssen, 2006). The *Acidobacteria* might also be metabolically active as well as numerically dominant in soils (Lee et al., 2008). They exhibit slow metabolic rates under low nutrient conditions and are versatile heterotrophs (Ward et al., 2009) and oligotrophs (Nemergut et al., 2010). The low soil pH value of this study site also facilitates the presence of *Acidobacteria*, and their distribution is negatively correlated with soil pH (Jones et al., 2009). According to the genomic and physiological data, *Acidobacteria* also well tolerate fluctuations in soil hydration (Ward et al., 2009). Moreover, some studies showed that *Acidobacteria* involve degradation of plant- and microorganism-based polysaccharides and are linked to the soil N availability (Fierer et al., 2007; Jones et al., 2009). In the present study, the abundant acidobacterial Gp1 and Gp2 were also reported as the two most abundant subdivisions of *Acidobacteria* in the tundra soil bacterial communities. They responded to environmental conditions differently, suggesting the wide functional diversity of these organisms (Männistö et al., 2013). Hence, based on their abundance, metabolic activity, and the presence in various soils, the *Acidobacteria* appear to play important roles in this forest ecosystem as well as in other environments.

*Proteobacteria* were abundant in the three soil communities. Several studies in other soils showed similar trends (Mendes et al., 2015; Montecchia et al., 2015). Members of this phylum possess an enormous range of morphological, physiological, and metabolic diversity and play essential roles in global carbon, nitrogen, and sulfur cycling (Spain et al., 2009). Among *Proteobacteria*, many alphaproteobacterial pyrosequences were related to the genus *Bradyrhizobium* of the *Rhizobiales*. Some species related to this genus are able to fix nitrogen and could contribute to the biogeochemical cycling in this ecosystem (Zhang and Xu, 2008; Yarwood et al., 2009). In addition, the members of *Rhizobiales* are considered a rhizospheric-plant promoting group (Fierer et al., 2007). Their abundance could indicate the essential roles in plant–microbe interactions and soil function, as well as in nitrogen fixation. Within *Betaproteobacteria*, the genus *Burkholderia* was also abundant and relatively higher in the winter communities. This group is a commonly part of rhizosphere soil communities. With functional diversity in nitrogen fixation and plant growth promotion (Coenye and Vandamme, 2003), it could play important role in maintaining stability in the soil community.

In the three communities, *Actinobacteria* accounted for less than 6% of the sequences. The abundance of *Actinobacteria* increases in soils with high organic matter and alkaline pH (Zhang et al., 2013; Li et al., 2016). The acidic pH value (pH < 4) of the study soils could result in lower abundance in *Actinobacteria*, since their relative abundance is positively correlate with soil pH (Lauber et al., 2009).

The phylum *Chloroflexi* also comprised about 3–4% of the three communities, with a relatively higher abundance in CONIF-40 soils. The species of this phylum are able to hydrolyze polysaccharides such as cellulose, xylan, and chitin (Yabe et al., 2010, 2016), suggesting their roles in degradation of plant residue.

## Conclusion

Though similar in composition, the bacterial structure among forest types and seasons were significantly different. The composition of the communities differed, especially between seasons. The bacterial diversity was also higher in the community with a shorter reforestation time and in summer, except that in BROAD-Nat. The differences between two seasons could have resulted from differences in soil moisture and temperature. More environmental factors would need to be examined to completely elucidate the relationship between soil properties and soil bacterial communities and the effects of season on the soil bacterial communities at this site.

## Author Contributions

Y-TL performed statistical analyses and wrote the manuscript. WW helped in analyzing and interpreting the data. WW, DC, and C-YC edited the manuscript. All authors read and approved the final manuscript.

## Conflict of Interest Statement

The authors declare that the research was conducted in the absence of any commercial or financial relationships that could be construed as a potential conflict of interest.

## Footnotes

1 http://pyro.cme.msu.edu
