# Supplementary material for: Effects of Reforestation on the Structure and Diversity of Bacterial Communities in Subtropical Low Mountain Forest Soils
Source: Front Microbiol. 2018 Aug 21;9:1968. doi: 10.3389/fmicb.2018.01968 (PMC6110939; doi:10.3389/fmicb.2018.01968)
Supplement: Supplementary file 1 [file Data_Sheet_1.docx]

**Table S1.** Number of reads obtained from three forest soil bacterial communities.

|  | Summer |  |  |  | Winter |  |  |
| --- | --- | --- | --- | --- | --- | --- | --- |
|  | BROAD-Nat | CONIF-80 | CONIF-40 |  | BROAD-Nat | CONIF-80 | CONIF-40 |
| No. of reads | 20308 | 23731 | 24060 |  | 14713 | 12341 | 18264 |

**Table S2.** Statistical analysis of differences among forest soil communities with K_CF_ from *K*-shuff analysis. Values (mean ± SE) in bold are *P* *≤* 0.05.

|  | BROAD-Nat | CONIF-80 | CONIF-40 | All |
| --- | --- | --- | --- | --- |
| Seasons | **0.0131±0.0015** | **0.0133±0.0002** | **0.0157±0.0034** | **0.0139±0.0026** |
| Summer | 0.0142±0.0009 | 0.0149±0.0003 | 0.0181±0.0039 | **0.0157±0.0019** |
| Winter | 0.0120±0.0002 | 0.0117±0.0012 | 0.0133±0.0011 | **0.0123±0.0005** |
| Replicates | 0.0132±0.0014 | 0.0134±0.0020 | 0.0159±0.0040 | 0.0142±0.0029 |

**Supplementary figure caption**

**FIGURE S1.** Relative abundance of bacterial phyla among three forest soil bacterial communities.

**FIGURE S2.** Venn diagrams of the percent of shared OTUs comprising 10 or more reads among (a) the three soil bacterial communities in summer and winter and each community (b) BROAD-Nat, (c) CONIF-80 and (d) CONIF-40 in different seasons. OTUs were formed at an evolutionary distance of 3 %. The total number of OTUs was (a) 286 and 273 in summer and winter, respectively, (b) 133, (c) 174 and (d) 186. The total reads included for the Venn diagram were about 60-65% of normalized data in a, and about 53-57% in b-d.

**FIGURE S3.** Rarefaction curves analysis with all sequence data of the three forest soil bacterial communities. OTUs were calculated at the 3 % evolutionary distance.

**FIGURE S1.**


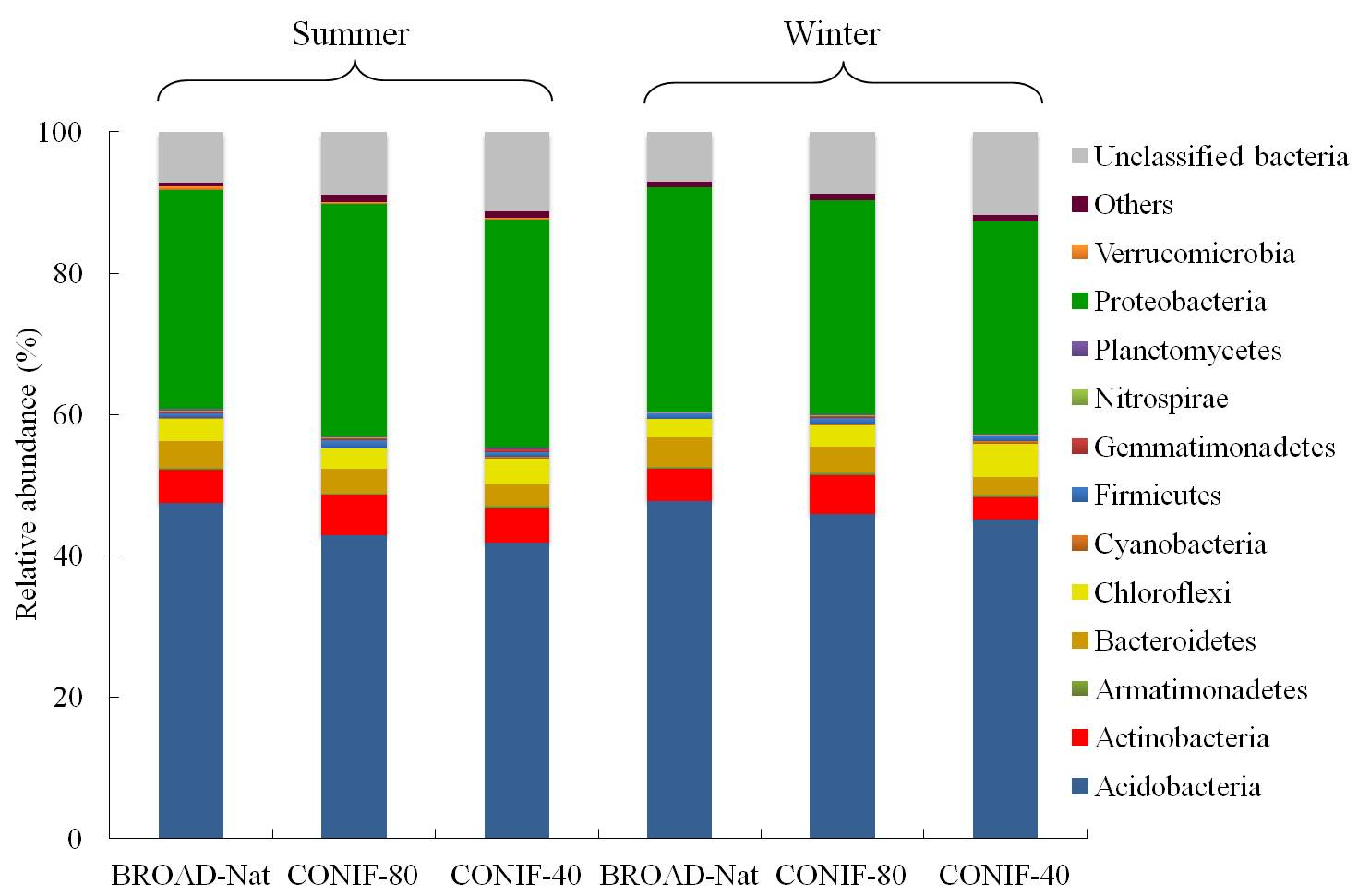


**FIGURE S2.**


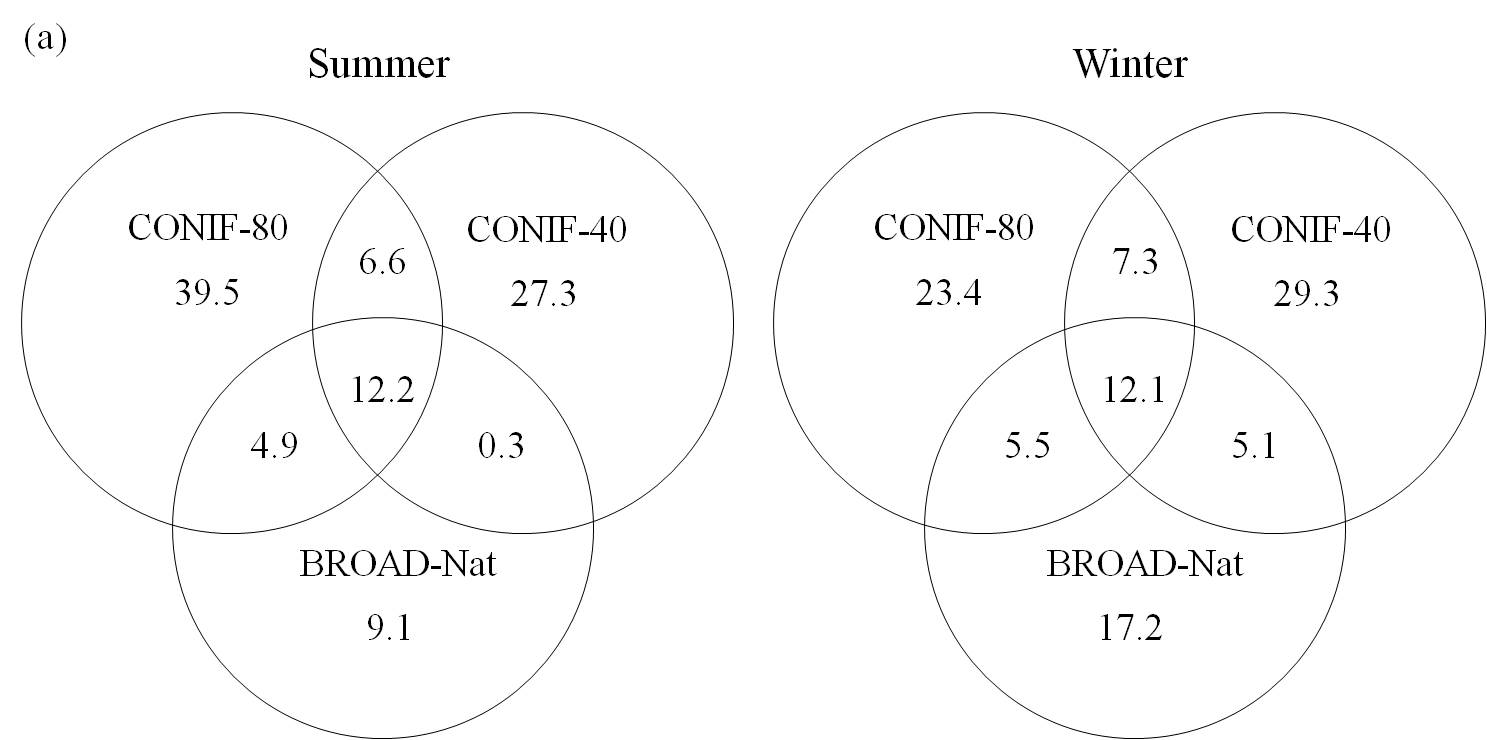


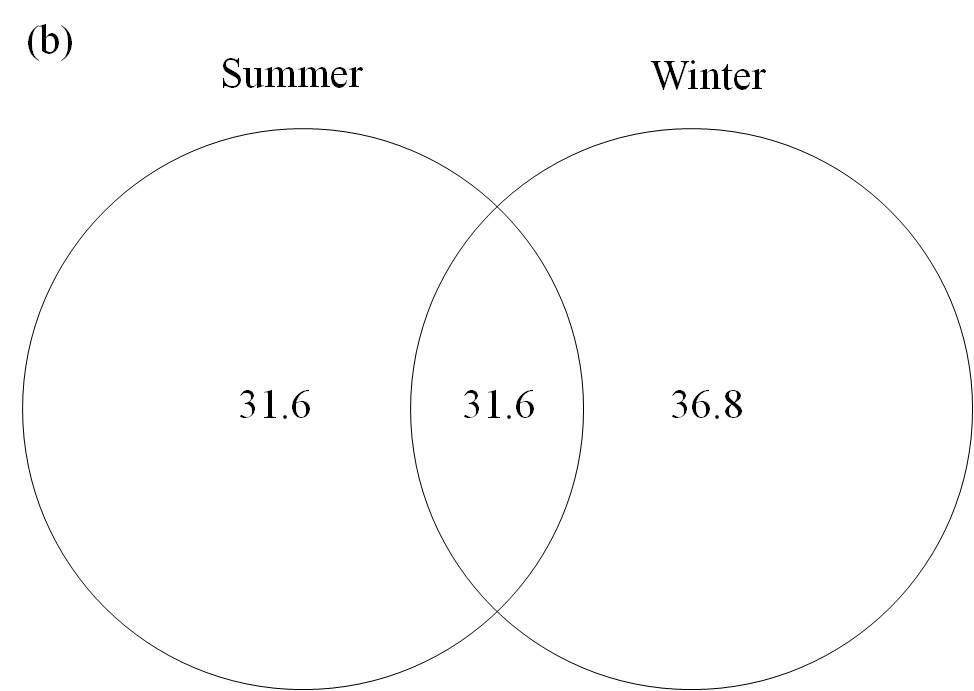


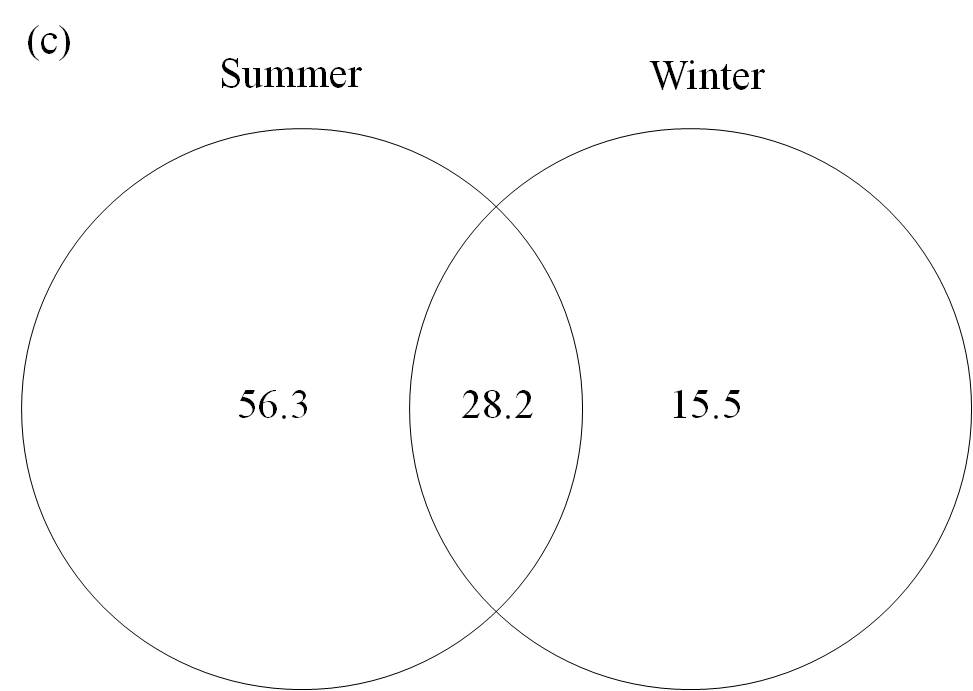


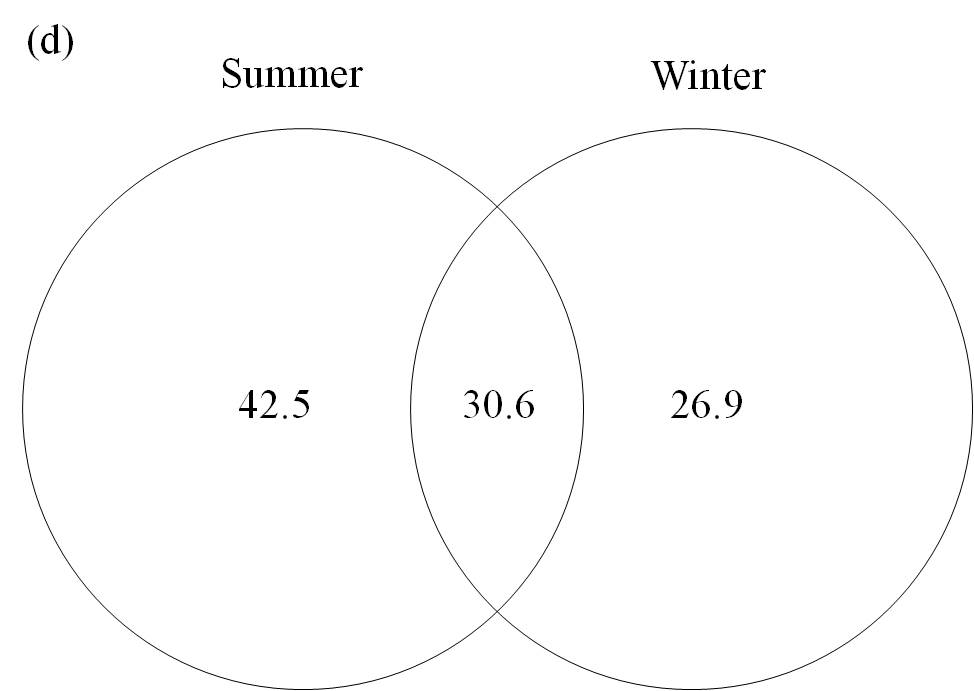


**FIGURE S3.**
